# The Histone Variant H3.3 Is Enriched at *Drosophila* Amplicon Origins but Does Not Mark Them for Activation

**DOI:** 10.1534/g3.116.028068

**Published:** 2016-04-06

**Authors:** Neha P. Paranjape, Brian R. Calvi

**Affiliations:** Department of Biology, Indiana University, Bloomington, Indiana 47405

**Keywords:** DNA replication, H3.3, amplicon, chromatin, histone variant

## Abstract

Eukaryotic DNA replication begins from multiple origins. The origin recognition complex (ORC) binds origin DNA and scaffolds assembly of a prereplicative complex (pre-RC), which is subsequently activated to initiate DNA replication. In multicellular eukaryotes, origins do not share a strict DNA consensus sequence, and their activity changes in concert with chromatin status during development, but mechanisms are ill-defined. Previous genome-wide analyses in *Drosophila* and other organisms have revealed a correlation between ORC binding sites and the histone variant H3.3. This correlation suggests that H3.3 may designate origin sites, but this idea has remained untested. To address this question, we examined the enrichment and function of H3.3 at the origins responsible for developmental gene amplification in the somatic follicle cells of the *Drosophila* ovary. We found that H3.3 is abundant at these amplicon origins. H3.3 levels remained high when replication initiation was blocked, indicating that H3.3 is abundant at the origins before activation of the pre-RC. H3.3 was also enriched at the origins during early oogenesis, raising the possibility that H3.3 bookmarks sites for later amplification. However, flies null mutant for both of the H3.3 genes in *Drosophila* did not have overt defects in developmental gene amplification or genomic replication, suggesting that H3.3 is not essential for the assembly or activation of the pre-RC at origins. Instead, our results imply that the correlation between H3.3 and ORC sites reflects other chromatin attributes that are important for origin function.

DNA replication in multicellular eukaryotes initiates from multiple origins to ensure timely duplication of the genome (Huberman 1968; Mechali 2010). The right number of origins must be activated exactly once every cell cycle to avoid either under-replication or over-replication of the genome. Defects in origin regulation compromise genome integrity, contribute to cancer, and cause heritable developmental syndromes (Negrini *et al.* 2010; Bicknell *et al.* 2011a, 2011b; Guernsey *et al.* 2011; Kawabata *et al.* 2011; Hills and Diffley 2014). Despite the importance of origin regulation, much remains unknown about how certain regions of the genome are selected to be active origins. While it is clear that origin activity is strongly influenced by chromatin, molecular mechanisms are not fully defined (Mechali *et al.* 2013; Hyrien 2015). Here, we investigate how the histone variant H3.3 influences origin function in *Drosophila*.

The proteins that are required for origin function are conserved from yeast to humans, but, in metazoa, little is known about how specific genomic sites are selected to be origins (Remus and Diffley 2009; Mechali 2010). The origin recognition complex (ORC) binds to origin DNA during G1-phase, and serves as a platform for assembly of a prereplicative complex (pre-RC). A subset of these pre-RCs are then activated by S-phase kinases, resulting in DNA helix unwinding and the recruitment of other proteins to establish active replication forks (Diffley *et al.* 1995; Fernandez-Cid *et al.* 2013; Frigola *et al.* 2013; Weinreich 2015; Yeeles *et al.* 2015). In recent years, genomic approaches have identified thousands of origins in a variety of organisms including humans (Aladjem 2007; MacAlpine *et al.* 2010; Cayrou *et al.* 2011; Eaton *et al.* 2011; Gilbert 2012). Although some DNA sequence signatures have been reported, a strict origin consensus sequence has not emerged (Vashee *et al.* 2003; Remus *et al.* 2004; Cayrou *et al.* 2011, 2012; Besnard *et al.* 2012; Comoglio *et al.* 2015; Foulk *et al.* 2015). Thus, while it is clear that pre-RC assembly and activation occurs at specific sites in the genome, what specifies these sites remains incompletely defined.

It is now evident that chromatin exerts a strong influence on origin activity. Changes to chromatin during development correlate with different origin activities among cells (Hiratani *et al.* 2010; Besnard *et al.* 2012). Origins that are highly active, and that replicate early in S phase, tend to reside in genomic domains with active genes, and are enriched for histone acetylation and other activating chromatin modifications (Aggarwal and Calvi 2004; Danis *et al.* 2004; Hiratani and Gilbert 2009; MacAlpine *et al.* 2010; Unnikrishnan *et al.* 2010; Eaton *et al.* 2011; Liu *et al.* 2012). Conversely, origins within heterochromatin are less active and replicate later in S phase, although there are exceptions to this rule (Kim *et al.* 2003; Hayashi *et al.* 2009; Schwaiger *et al.* 2010). In some cases, how specific types of chromatin modifications influence pre-RC assembly and activation have been described, including the dimethylation of lysine 20 on histone H4 (H4K20me2), and acetylation of multiple lysines on histones H3 and H4 (Iizuka and Stillman 1999; Aggarwal and Calvi 2004; Miotto and Struhl 2008, 2010; Tardat *et al.* 2010; Kuo *et al.* 2012; Liu *et al.* 2012; McConnell *et al.* 2012). The position of nucleosomes also impacts origins, with most ORC binding sites being depleted of nucleosomes, whereas nucleosomes adjacent to some origins may actually assist pre-RC assembly (Lipford and Bell 2001; Eaton *et al.* 2010, 2011; MacAlpine *et al.* 2010; Muller *et al.* 2010; Hoggard *et al.* 2013; Liu *et al.* 2015). Despite these advances, the molecular mechanisms by which chromatin impacts origins remain little understood.

Genome-wide studies have shown that another chromatin property that correlates with origins is the histone variant H3.3 (Deal *et al.* 2010; MacAlpine *et al.* 2010; Eaton *et al.* 2011; Stroud *et al.* 2012). This correlation has led to speculation that nucleosomes containing H3.3 are important for marking sites of pre-RC assembly or activation (Deal *et al.* 2010; MacAlpine *et al.* 2010). The H3.3 variant differs from canonical H3 histones (H3.1 and H3.2) by just four to five amino acids depending on organism (Elsaesser *et al.* 2010; Szenker *et al.* 2011). Unlike canonical histone genes, which are present in high copy number, there are only two H3.3 genes in most multicellular eukaryotic genomes, including human, mouse, and *Drosophila* (Elsaesser *et al.* 2010; Szenker *et al.* 2011). While canonical H3 expression, and its incorporation into chromatin, is limited to S phase, the H3.3 variant is expressed throughout the cell cycle, and is deposited into chromatin in both a replication-dependent and independent manner (Ahmad and Henikoff 2002; Szenker *et al.* 2011). Nucleosomes that contain the two-histone variants H3.3 and H2A.z (H2Av in *Drosophila*) have a dynamic association with DNA, and are enriched at enhancers, promoters, and other highly accessible and active regions of the genome (Jin and Felsenfeld 2007; Henikoff 2009; Jin *et al.* 2009; Talbert and Henikoff 2010). One possibility is that loci containing these dynamic nucleosomes permit assembly of the pre-RC onto DNA, explaining the correlation of H3.3 with origins. Mutations or altered levels of H3.3, as well as the proteins required for its chromatin deposition, are drivers of pediatric and adult glioblastoma multiforme (GBM) (Schwartzentruber *et al.* 2012; Wu *et al.* 2012; Gallo *et al.* 2015). Given the correlation between H3.3 and origins, the high level of genome instability in these GBM tumors may, in part, be a manifestation of origin dysfunction. Thus, it is crucial to determine whether H3.3 plays a functional role at origins.

In this study, we use developmental gene amplification in somatic follicle cells of the *Drosophila* ovary as a model to evaluate the function of H3.3 at origins. During amplification, rereplication from origins at six specific loci increases the copy number of genes that are required for egg shell synthesis (Spradling 1981; Calvi 2006; Kim *et al.* 2011; Liu *et al.* 2012). These origins are genetically and molecularly well-defined, and provide a number of methodological advantages. Previous evidence indicated that nucleosome acetylation and position are important for ORC binding and amplicon origin function (Aggarwal and Calvi 2004; Hartl *et al.* 2007; Kim *et al.* 2011; Liu *et al.* 2012, 2015; McConnell *et al.* 2012). Here, we show that H3.3 is highly enriched at amplicon origins, beginning early in oogenesis before amplification, consistent with the idea that dynamic H3.3 nucleosomes may bookmark these sites for later amplicon origin activity. However, analysis of null mutants for histone H3.3 showed no overt defects in amplicon origin activity or genomic DNA replication, suggesting that this histone variant is not essential for the activity of most origins. Although not required for origin function, our results suggest that H3.3 abundance is associated with a chromatin environment that is conducive to origin activity.

## Materials and Methods

### Fly strains and genetics

All flies were raised at 25° on standard media. The heat shock inducible *HS-H3.3A-GFP* and *HS-H3-GFP* (Ahmad and Henikoff 2002), and H3.3 null mutants H3.3B^0^;H3.3A^2*1^/CyO-GFP (Sakai *et al.* 2009) were generous gifts from Kami Ahmad. The two strains with deficiencies encompassing the *H3.3A* gene were *w^1118^*; *Df(2L)Exel7022 / CyO* and *w^1118^*; *Df(2L)BSC110 / CyO*, and were obtained from the Bloomington *Drosophila* stock center. The *C323:Gal4* was used to drive the expression of *P{ w^+mC^ UAS:dacapo}* in flies that also had the heat inducible *HS-H3.3A-GFP* (de Nooij *et al.* 1996; Lane *et al.* 1996). Heat induction of *HS-H3.3A-GFP* was at 37° for 30 min, followed by recovery for 90 min at 25° before fixation for immunostaining or ChIP.

### Antibodies, immunostaining, and microscopy

BrdU, antibody and DAPI labeling for *Drosophila* ovaries were performed as previously described (Calvi and Lilly 2004). α-BrdU (mouse monoclonal, BDB347580; BD Biosciences, San Diego, CA) was used at 1:20. α-GFP (rabbit, Molecular Probes, Life Technologies), and secondary antibodies Alexa 488 anti-rabbit (Invitrogen) were used at 1:500. For EdU labeling, unfixed cells were incubated in 10 µM EdU followed by fixation with formaldehyde. EdU incorporation was detected with Alexa Fluor-488 Azide (Invitrogen) in a copper-catalyzed Click-iT reaction (Buck *et al.* 2008; Salic and Mitchison 2008). Images were taken with a Leica DMRA2 widefield epifluorescence microscope (Figure S2, and Figure S5, C and D), and a Leica SP5 laser scanning confocal microscope (Figure 2, Figure 6, Figure S1, Figure S4, and Figure S5, A and B).

### Follicle cell nuclear preparation

Follicle cell nuclei were purified from different stage egg chambers as described previously (Liu *et al.* 2012). Conditioned young adult females were blended by short pulses in cold phosphate-buffered saline buffer in 0.02% Tween-20 in a household blender. Released egg chambers were enriched by serial filtration through 250- to 70-μm meshes. Eggs were then fixed for 15 min at room temperature in 2% formaldehyde solution, followed by quenching with 125 mM glycine, and washing with cold Dulbecco’s phosphate-buffered saline. Stage 1–8 and stage 10 egg chambers were then further manually purified from the crude fractions, and stored at −80° prior to nuclear preparation. Approximately 600–700 stage 10 egg chambers were used for each nuclear preparation. For stage ≤ 8 egg chambers, a tissue volume similar to that of the stage 10 was used. Frozen eggs were thawed on ice, resuspended in mHB buffer (0.34 M sucrose, 15 mM NaCl, 60 mM KCl, 0.2 mM EDTA, 0.2 mM ethylene glycol tetraacetic acid, 0.15 mM spermine, 0.15 mM spermidine in 15 mM Tris-HCl, pH 8.0) supplemented with 0.5% NP-40. The tissue was homogenized in a Kontes 2-ml douncer via 15 strokes with a type A pestle. The lysate was filtrated with a 15-μm Nytex nylon membrane to remove the larger nurse cell nuclei. The filtrate was spun 3 min at 500 × *g* to pellet follicle nuclei.

### Chromatin immunoprecipitation

The chromatin immunoprecipitation (ChIP) protocol was as previously described, with slight modifications, and entailed biological replicates from separate isolations of follicle cell nuclei (Liu *et al.* 2012). Briefly, the prepared follicle cell nuclei were resuspended in nuclear lysis buffer (1% SDS, 1 mM EDTA in 50 mM Tris-Cl, pH 8.0), and subjected to sonication [Diagenode (Bioruptor sonication device), tip probe, high setting, four rounds of 5 min (30-sec on/off) sonication on iced water bath] to a modal size of 450 bp. One-sixth of the sample was saved as input, and the remainder was aliquoted and diluted at least five-fold into IP dilution buffer (0.01% SDS, 1.1% Triton-X 100, 1.1 mM EDTA, 167 mM NaCl in 20 mM Tris-Cl, pH 8.0) to a final volume of 1 ml. H3.3A-GFP ChIP was performed using 2 µl (∼2 µg) of rabbit polyclonal GFP antibody (Molecular Probes, Life Technologies), and Orc2 ChIP used a rabbit polyclonal Orc2 antibody (Austin *et al.* 1999); normal rabbit serum (1 mg/ml, Jackson ImmunoResearch) was used as a negative control. Incubation with antibody was with nutation at 4° overnight; 30 μl of 50% protein A agarose beads (15918-014; Invitrogen) pretreated with sheared salmon sperm DNA and BSA were added and incubated 1–3 hr at 4° to pull down the chromatin bound to the antibody. The beads were washed two times each with low-salt, high-salt, LiCl, and Tris–EDTA buffers. Elution and reversal of cross-linking was done by heating at 65° overnight with 5 M NaCl in nuclear lysis buffer. Both the input and eluate were treated with 1 μg of RNaseA (11119915001; Roche, Indianapolis, IN), and 50 μg of proteinase K (P8102S; New England BioLabs, Ipswich, MA). DNA was then purified with standard phenol/chloroform procedure and used for qPCR analysis.

### qPCR

The enrichment of H3.3A-GFP or ORC at different *Drosophila* Amplicon in Follicle Cells (DAFCs) was determined by qPCR. Forty cycles of a two-step PCR protocol (denaturation at 95° for 15 sec, and annealing/extension at 62° for 30 sec) were run. The analysis was done on a Stratagene (Santa Clara, CA) Mx3005P machine with SYBR Green Master Mix (600843; Agilent, Santa Clara, CA). For qPCR quantification, the amount of DNA in the pellet as expressed as percentage of input DNA estimated by a standard curve generated from a serial dilution of the input. The values were then normalized to two control, nonorigin, loci at cytogenetic position 64A and 93E/F or to 93E/F alone (Figure 4). Enrichment was also determined at the *Drosophila hsp70* locus, as a positive control (Schwartz and Ahmad 2005).

### Statistical analysis

H3.3A-GFP enrichment at DAFC-66D and other amplicons DAFC—7F, 22B, 30B, 34B and 62D was measured as % input in stage 1–8 and stage 10 follicle cell nuclei, and compared to H3.3A-GFP enrichment in those stages at the nonorigin control loci 64A and 93E/F. H3.3A-GFP enrichment at DAFC-66D and other amplicons was also compared in the presence and absence of CDK inhibitor *dacapo*. The *P*-values for difference in enrichment over multiple biological replicates was computed by Ratio Paired *t*-Test using GraphPad Prism version 6.0 for Windows, GraphPad Software (La Jolla, CA; www.graphpad.com).

### Data availability

The authors state that all data necessary for confirming the conclusions presented in the article are represented fully within the article. All data and reagents are available upon request.

## Results

### H3.3 is enriched at the active amplicon origins

**Figure 1:**
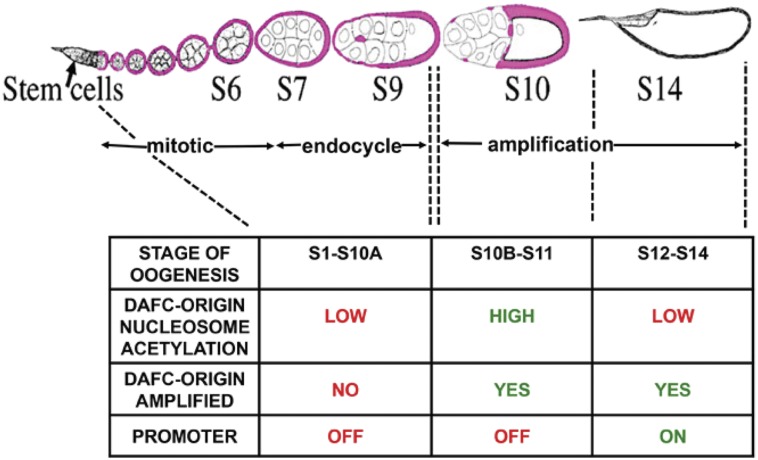
Developmental timeline of *Drosophila* oogenesis, follicle cell cycles, and amplicon origin activity. Egg chambers mature through 14 stages as they migrate posteriorly in the ovariole (from left to right). Somatic follicle cells (pink) surround the germline nurse cells and oocyte in each egg chamber. Follicle cells have three types of DNA replication programs: mitotic cycle (stages 1–6), endocycle (stages 7–10A), and developmental gene amplification (stages 10B–14). The table below shows the status of the amplicon origin, chorion gene promoters, and chromatin at DAFC-66D during different cell cycle programs.

The *Drosophila* ovary affords a number of advantages for evaluating H3.3 function during DNA replication. The ovarian somatic follicle cells form an epithelial sheet around egg chambers, and undergo three types of DNA replication programs (Figure 1). These DNA replication programs are coordinated with maturation of the egg chambers as they develop through 14 stages as they migrate down the ovariole (Figure 1). Follicle cells proliferate via the canonical mitotic cycle in stages 1–6, periodically duplicate their genome without division during three endocycles (G/S cycle) in stages 7–10A, and then undergo developmental gene amplification in stages 10B–14 (Figure 1) (Calvi and Spradling 1999; Claycomb and Orr-Weaver 2005; Horne-Badovinac and Bilder 2005; Calvi 2006). During gene amplification, genomic replication ceases, and amplicon origins initiate multiple rounds of DNA replication at six loci, resulting in an increase in the DNA copy number of genes required for eggshell synthesis (Spradling 1981; Calvi *et al.* 1998; Kim *et al.* 2011).

**Figure 2:**
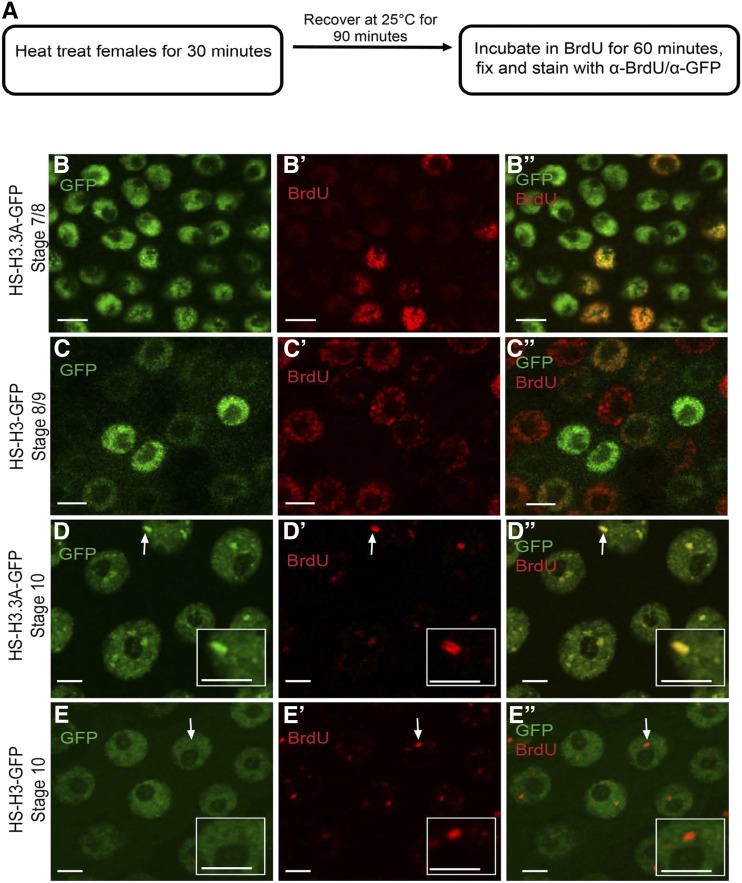
H3.3 is enriched at the amplicon origins. (A) Experimental scheme for heat induction of H3.3A-GFP and BrdU labeling of follicle cells. (B)–(E”) Anti-GFP (B, C, D, and E), BrdU (B’, C’, D’, and E’), and merged images (B”, C”,D”, and E”) in endocycling (B–C”) and stage 10B amplifying (D–E”) follicle cells that are expressing either the histone variant H3.3A-GFP (B–B” and D–D”) or the canonical histone H3-GFP (C–C” and E–E”), using the protocol described in (A). White arrows in D–D” and E–E” indicate one highly-amplified DAFC-66D locus, which is shown at higher magnification in the insets. The BrdU double bars in D’, D” represent replication forks migrating bidirectionally outward from the origin. See Figure S1 for quantification. Scale bars are 5 µm.

To evaluate the behavior of H3.3 during different types of replication programs, we used a fly strain in which one of the two identical H3.3 genes (*H3.3A*) is fused to GFP and under the control of the heat-inducible hsp70 promoter (*HS-H3.3A-GFP*) (Ahmad and Henikoff 2002). We compared it to canonical histone H3 that was also fused to GFP and heat-inducible (*HS-H3-GFP*). Expression of these transgenes was induced in adult females at 37° for 30 min. Females were allowed to recover for 90 min before ovaries were dissected and incubated in the nucleotide analog BrdU for 60 min, followed by immunofluorescent labeling for BrdU and GFP (Figure 2A).

During mitotic cycles and endocycles (stages 1–10A), both H3.3A-GFP and H3-GFP labeled throughout the nuclei of a subset of follicle cells (Figure 2, B-C”). During amplification stages 10B-13, both H3.3A-GFP and H3-GFP had a low level of pan-nuclear, focal GFP labeling (Figure 2, D and E). In addition, stage 10B-13 follicle cells had several large nuclear foci of H3.3A-GFP, but not H3-GFP (Figure 2, D and E). We wondered whether the larger H3.3A-GFP foci corresponded to amplifying gene loci. During these stages, developmental gene amplification can be seen as foci of BrdU incorporation (Figure 2, D’ and E’) (Calvi *et al.* 1998; Calvi and Lilly 2004). The H3.3A-GFP foci colocalized with BrdU foci, whereas canonical H3-GFP did not (Figure 2, D-E”, and Figure S1, A-B’). These results suggest H3.3A-GFP is dynamically incorporated into chromatin at active amplicons to levels that exceed that of canonical H3-GFP.

To evaluate H3.3A-GFP incorporation at higher resolution, we analyzed its pattern at the largest BrdU focus. This focus is DAFC-66D, the amplicon that amplifies to highest copy number (∼64-fold), and which is the best characterized (Figure 2D”) (Spradling 1981). At DAFC-66D, ORC is bound during stages 10B-12—a time when origin nucleosomes are hyperacetylated and the origin is active—but the activity is low for nearby promoters of eggshell protein (chorion) genes (Figure 1) (Griffin-Shea *et al.* 1982; Aggarwal and Calvi 2004; Cavaliere *et al.* 2008; Kim *et al.* 2011; Liu *et al.* 2012). Since the chorion genes at DAFC-66D are not highly expressed during stages 10-11, most of the incorporation of H3.3A-GFP at this time is not mediated by transcription (Griffin-Shea *et al.* 1982; Cavaliere *et al.* 2008).

Previous studies have shown that immunolabeling at DAFC-66D has sufficient resolution to distinguish the location of proteins at origins or forks (Loebel *et al.* 2000; Whittaker *et al.* 2000; Calvi and Spradling 2001; Claycomb *et al.* 2002; Nordman *et al.* 2014; Alexander *et al.* 2015). During stage 10, the H3.3A-GFP foci had an extended morphology of ∼1.5 µm that was both between, and coincident with, double bars of BrdU incorporation, which represent forks migrating outward from the origin (Figure 2, D-D”, and Figure S1, A and A’). In contrast, fluorescence quantification indicated that the occupancy of canonical H3-GFP at DAFC-66D origins and forks was not different than other nuclear regions (Figure S1B and B’). In stage 12, ORC departs, the origin shuts off, and nearby chorion gene promoters become active (Figure 1) (Austin *et al.* 1999; Royzman *et al.* 1999). During this stage, H3.3A-GFP was again distributed both between, and coincident with, the double bars of nucleotide incorporation at the replication forks that continue to migrate outwards from the quiescent origin (Calvi and Spradling 2001) (Figure S1, C and C’, File S1). Comparing the size of the H3.3A-GFP foci to previous multi-color FISH measurements at DAFC-66D suggested that H3.3A-GFP incorporation occurs over a region of at least ∼20 kb, and is not restricted to DAFC-66D DNA replication initiation sites (Calvi and Spradling 2001).

### H3.3 is abundant across multiple sites at DAFC-66D

**Figure 3:**
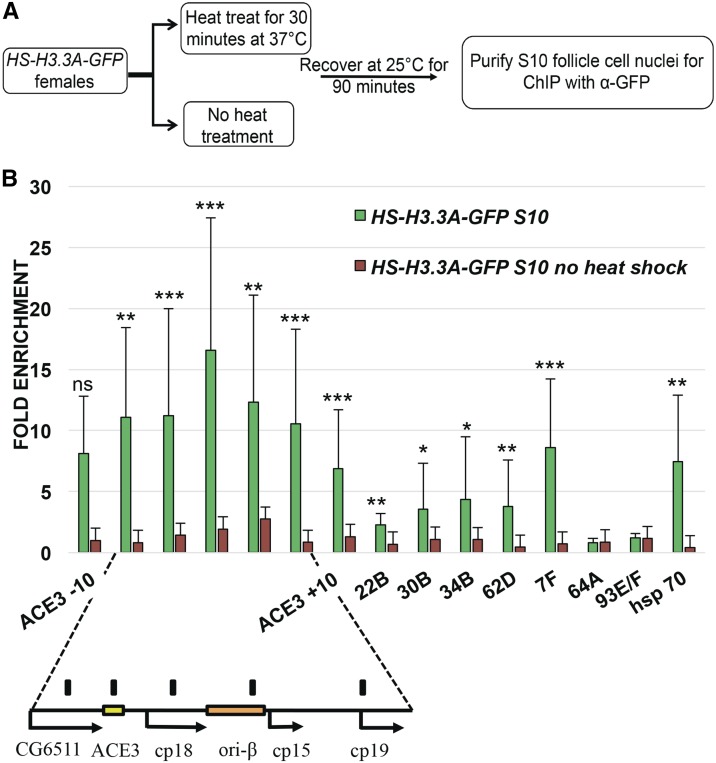
H3.3 is enriched at amplicon origins. (A) Experimental scheme for H3.3A-GFP ChIP- qPCR for amplification stage 10 follicle cells. (B) Anti-GFP ChIP-qPCR analysis of stage 10 follicle cell nuclei after heat induction of H3.3A-GFP (green bars) or no heat induction control (small orange bars). The *y*-axis represents fold enrichment of H3.3A-GFP in the pellet relative to input, and then normalized to two nonorigin negative control loci, 93E/F and 64A. H3.3A-GFP deposition into the induced hsp70 transcribed region was a positive control. The DAFC-66D amplicon is shown below, including the essential regions ACE3 and ori-β. Multiple primers were used to quantify H3.3A-GFP chromatin across DAFC-66D, as well as 10 kb to either side of ACE3 (ACE3 –10 and ACE3 +10). The position of these qPCR primers at DAFC-66D are represented by black bars above the map. Also shown is the enrichment of H3.3A-GFP at the other amplicons DAFC—7F, 22B, 30B, 34B, and 62D. H3.3A-GFP was significantly enriched at multiple loci across DAFC 66D and other DAFCs in stage 10 follicle cells compared to the 64A and 93E/F control loci. (* *P* < 0.05, ** *P* < 0.01, *** *P* < 0.001 by ratio paired *t*-test, see Table S1). The data for heat induced H3.3A-GFP represent an average of at least three biological replicates, while values for no heat induction negative controls represent an average of at least two biological replicates. The error bars represent the range of values.

We then used anti-GFP antibodies for ChIP-qPCR to quantify the abundance of H3.3A-GFP at the amplicons to high-resolution. H3.3A-GFP expression was heat induced for 30 min, or not induced in controls, and, 1.5 hr later, stage 10 egg chambers were isolated, formaldehyde fixed, and follicle cell nuclei purified away from larger nurse cell nuclei by filtration, resulting in a high developmental resolution (Figure 3A). Cross-linked follicle cell chromatin was then sheared, and H3.3A-GFP immunoprecipitated with anti-GFP antibodies (Figure 3A). The amount of DAFC-66D DNA in the input and pellet was then assessed by qPCR, which internally controls for developmental amplification of copy number. The input: pellet ratio at the amplicon was then normalized to the input: pellet ratio at two nonorigin control loci at cytogenetic positions 93E/F and 64A. The high-level incorporation of H3.3 during heat-induced transcription of the *hsp70*Ba locus served as a positive control (Schwartz and Ahmad 2005). The ChIP-qPCR results indicated that H3.3 is significantly enriched at multiple positions across the DAFC-66D locus in stage 10B follicle cells (Figure 3B and Table S1, Table S4). The levels of H3.3 at DAFC-66D (∼6- to 16-fold) were comparable to, or exceeded, the high levels of H3.3 incorporation at the hsp70 transcription unit (∼6-fold) (Figure 3B). H3.3 enrichment was highest in the middle of the DAFC-66D amplicon, coinciding with the two origin regions ACE3 and ori-β, and the 1.5-kb region between them (Figure 3B). ACE3 and ori-β are ORC binding sites, and are required for DAFC-66D origin function, with replication initiation occurring most frequently within ori-β (Delidakis and Kafatos 1988, 1989; Orr-Weaver *et al.* 1989; Heck and Spradling 1990; Lu *et al.* 2001). To either side of ACE3 and ori-β, there was a gradient of diminishing H3.3 occupancy spanning a total of ∼20 kb, consistent with the immunofluorescence measurements of H3.3A-GFP foci (Figure 3B and Figure S1, A, A’, C, and C’). In contrast, negative control H3.3A-GFP females that were not heat induced did not have detectable enrichment of DNA in the pellet (Figure 3B). These results suggested that H3.3 is abundant in chromatin across DAFC-66D when this origin is initiating DNA replication in stage 10 follicle cells.

We also evaluated H3.3A-GFP enrichment at five other amplicon loci (DAFC-7F, DAFC-22B, DAFC-30B, DAFC-34B, and DAFC-62D), which amplify to lower final DNA copy number (Kim *et al.* 2011) (Table S4). H3.3A-GFP was significantly enriched at these amplicon loci, but to a lesser extent than at DAFC-66D (Figure 3B). Among these other amplicons, the DAFC-7F locus amplifies most highly (∼16-fold DNA copy number), and was the most enriched for H3.3A-GFP (∼9-fold), while the amplicons that amplify 4- to 8-fold in DNA copy number had lower, but significant, enrichment of H3.3A-GFP (∼2- to 4-fold) relative to nonorigin control loci (Figure 3B and Table S1) (Claycomb and Orr-Weaver 2005; Kim *et al.* 2011). Thus, including DAFC-66D (∼64-fold amplified), there was a correlation between the abundance of H3.3 in origin chromatin and the number of times the origin initiates DNA replication. These data indicate that H3.3 is highly abundant in the chromatin of all the amplicons when these origins are initiating replication in stage 10.

### H3.3 deposition occurs before DNA replication initiates

**Figure 4:**
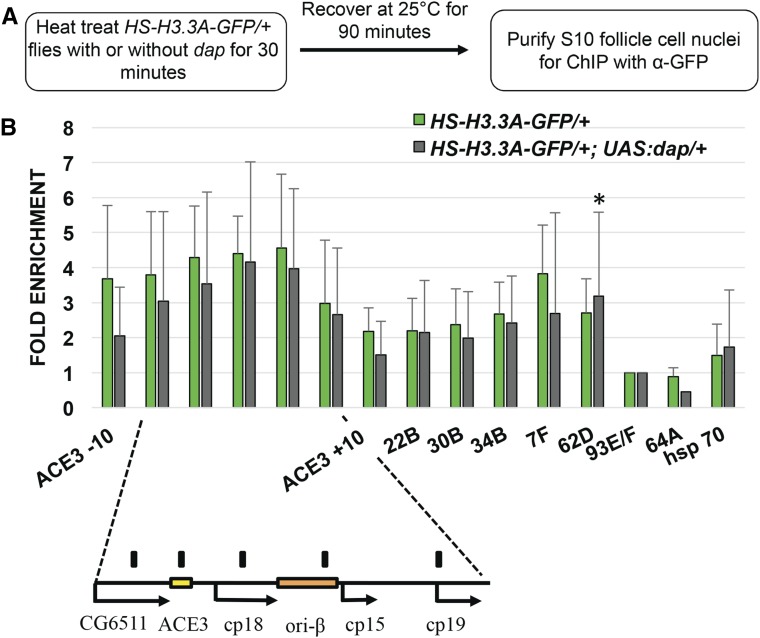
H3.3 is enriched at amplification origins before the initiation of DNA replication. (A) Experimental scheme for H3.3A-GFP ChIP-qPCR with or without *UAS:dacapo* (*UAS*:*dap*) expression. (B) H3.3 is still enriched at the amplicons when replication initiation is blocked by *dap*. Anti-GFP ChIP-qPCR analysis of stage 10 follicle cell nuclei after heat induction of *H3.3A-GFP* from flies with (gray bars) or without (green bars) coexpression of the CDK2 inhibitor *dap*. Ratio of DNA in the pellet *vs.* input was normalized to pellet/input ratio at negative control locus 93E/F. The hsp70 locus is a positive control. Note that, because the *H3.3A-GFP* transgene was hemizygous in these genetic crosses, H3.3A-GFP enrichment at the amplicons is lower than in previous experiments. The DAFC-66D amplicon and position of primers (black bars) are shown below. The other amplicons assayed were DAFC—7F, 22B, 30B, 34B and 62D. The occupancy of H3.3A-GFP was not significantly different from *UAS:dap* expression, except for DAFC-62D (* *P*-value of 0.04, as calculated by ratio paired *t*-test, see Table S2). Values represent the average of three biological replicates, and error bars represent SD.

In addition to its replication-independent deposition, it is known that H3.3 is deposited during chromatin assembly behind the replication fork (Ahmad and Henikoff 2002; Xu *et al.* 2010; Dunleavy *et al.* 2011; Frey *et al.* 2014). It remained unclear, therefore, whether the abundance of H3.3 at the amplicons represented replication-dependent incorporation, or whether H3.3 is also present at the origin before the initiation of DNA replication. To address this, we expressed the Cyclin E/CDK2 inhibitor *dacapo (dap*) beginning in stage 9 follicle cells, which blocks CDK activation of the pre-RC, but not pre-RC assembly, and strongly inhibits amplification (Figure 4A) (de Nooij *et al.* 1996; Lane *et al.* 1996; Calvi *et al.* 1998; Liu *et al.* 2012). We then compared the H3.3A-GFP ChIP-qPCR signal from cells hemizygous for H3.3A-GFP and that were, or were not, expressing *dap* (Figure 4B). The results showed that *dap* expression blocked replication but did not significantly reduce the H3.3A-GFP signal at the amplicons (Figure 4B and Figure S2). The distribution of H3.3A-GFP also appeared similar between dap-expressing and nonexpressing follicle cells, with highest abundance at the center of DAFC-66D (Figure 4B). These results suggest that much of the H3.3A-GFP incorporation at the amplicons occurs before the initiation of replication, and is not dependent on active replication forks.

### H3.3 is enriched at DAFC-66D origin early in oogenesis before amplification

**Figure 5:**
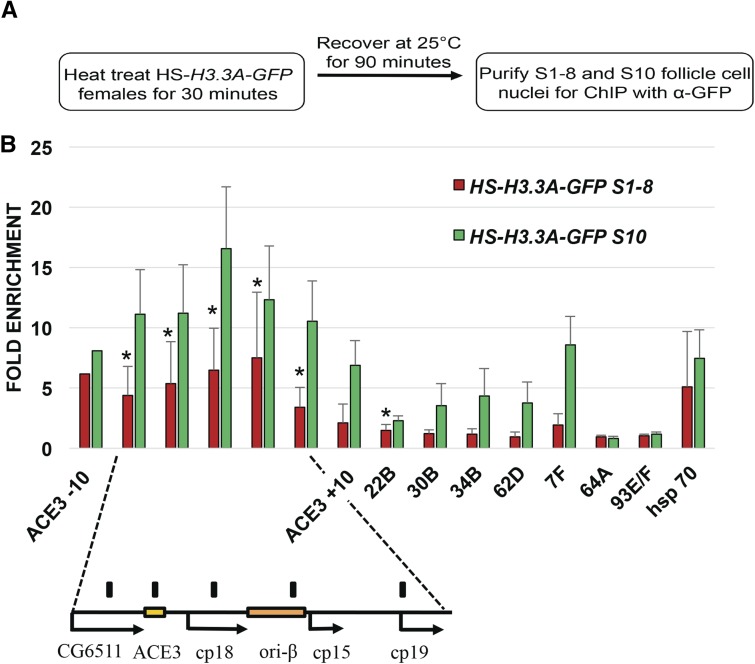
H3.3 is enriched at amplicon origins in early oogenesis before developmental amplification. (A) Experimental scheme for H3.3A-GFP ChIP-qPCR for stage 1–8 follicle cell nuclei. (B) Anti-GFP ChIP-qPCR analysis of stage 1–8 follicle cell nuclei after heat induction of *HS-H3.3A-GFP* (red bars). The relative abundance of *H3.3A-GFP* in stage 10 (green bars), when amplicon origins are active, was assessed in parallel from the same ovary preparations and is replotted from Figure 3B for comparison. During stages 1–8, H3.3A-GFP was significantly enriched at multiple loci across DAFC 66D and at DAFC 22B relative to the two nonorigin control loci 64A and 93E (* *P* < 0.05, ** *P* < 0.01 by ratio paired *t*-test). H3.3A-GFP occupancy was significantly lower during stages 1–8 than stage 10 (see Table S3 for *P*-values). Values represent average of at least three biological replicates, and error bars represent SD, except for ACE3 –10, which is based on two replicates.

The *dap* results indicated that H3.3A-GFP is present at the amplicons before activation of the pre-RC in stage 10 follicle cells. This raised the question as to whether H3.3 is enriched at the amplicons in follicle cells during early oogenesis before the onset of amplification. To address this, we repeated the H3.3A-GFP ChIP on stage 1–8 follicle cells, a time in oogenesis that is at least 24 hr before the onset of amplification (Calvi *et al.* 1998; Horne-Badovinac and Bilder 2005) (Figure 5A). The results indicated that H3.3A-GFP was significantly enriched at DAFC-66D relative to control loci in preamplification stage 1–8 follicle cells (∼4- to 6-fold), although to a lesser extent than in stage 10 (∼6- to 16-fold) (Figure 5B). Enrichment of H3.3A-GFP at the other amplicons was not significantly different from control loci during stages 1–8, except for DAFC-22B (Figure 5B). These data indicate that the deposition of H3.3 at the DAFC-66D origin begins during early oogenesis before the onset of amplification.

The combined evidence from follicle cells during stages 1–8, and those expressing *dap*, suggested that pre-RC activation is not required for H3.3 deposition at DAFC-66D. We wondered whether H3.3 is deposited at the origin before pre-RC assembly, the first step of which is binding of ORC to DNA (Mechali 2010). Several lines of evidence suggest that the activity of the DAFC-66D origin is specific to late stage follicle cells. Data from modENCODE indicated that DAFC-66D does not initiate DNA replication during the S phase of several *Drosophila* cell lines, and is not significantly occupied by ORC in cell lines, embryos, or larval salivary glands (MacAlpine *et al.* 2010; modENCODE Consortium*et al.* 2010; Eaton *et al.* 2011; Sher *et al.* 2012; Boley *et al.* 2014). Moreover, during the stage 9–10 transition at the onset of amplification, there is a significant increase in ORC occupancy at DAFC-66D, which is dependent on the acquisition of new histone acetylation at the origin (Austin *et al.* 1999; Aggarwal and Calvi 2004; Hartl *et al.* 2007; Kim *et al.* 2011; Liu *et al.* 2012; McConnell *et al.* 2012). However, the binding of ORC to DAFC-66D in stage 1–8 follicle cells has not been directly examined. To address this question, we used antibodies against the Orc-2 subunit for ChIP-qPCR on stages 1–8 and stage 10 follicle cells (Austin *et al.* 1999). While the mean ORC abundance was lower in stages 1–8 *vs.* stage 10, the variance in the data do not permit us to conclude that this difference is significant (Figure S3). Therefore, while all published evidence suggests that pre-RC binding and origin activity at DAFC-66D is specific to follicle cells in late oogenesis, our data do not permit us to conclude that H3.3 is bound before ORC in stages 1–8.

### H3.3 is not essential for genomic replication or developmental gene amplification

**Figure 6:**
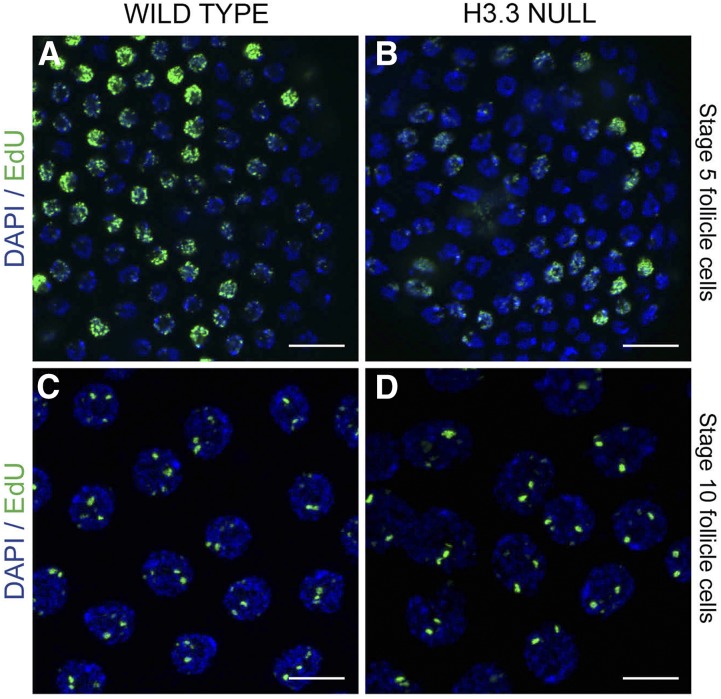
H3.3 is not essential for genomic DNA replication or developmental gene amplification. (A)–(D) Follicle cell nuclear DNA labeled with DAPI (blue) and for EdU incorporation (green) during genomic replication (A and B) and amplification (C and D) in wild type control (A and C) or H3.3 null females (B and D). The H3.3 null females were homozygous mutant for an H3.3B null allele on the X, and a null H3.3A allele over deficiency on the second, *H3.3B^0^*; H3.3A^2*1^ / *Df(2L)BSC110*. Scale bars are 10 µm.

The enrichment of H3.3 at DAFC-66D early in oogenesis raised the possibility that H3.3 may mark the locus for amplification during later oogenesis. This idea is consistent with the correlation between H3.3 and ORC binding sites from several genome-wide studies, which led to suggestions that H3.3 may be important for origin function (Deal *et al.* 2010; MacAlpine *et al.* 2010). To test this idea, we determined whether mutations in H3.3 impaired follicle cell genomic replication or developmental amplification. We used a strain that is null mutant for the two H3.3 genes in the *Drosophila* genome on the X and second chromosome (*H3.3B^0^* ; H3.3A^2*1^), which was previously reported to be homozygous semilethal (Sakai *et al.* 2009). To control for cryptic second site mutations, we crossed this strain to two different strains that have second chromosome deletions spanning the *H3.3A* gene [*Df(2L)Exel7022 and Df(2L) BSC110*]. For both crosses, most *H3.3B^0^*; H3.3A^2*1^/ Df(2L) null flies were lethal before adulthood, with only a few rare escaper adults that were male and female sterile. This lethality was not a result of cryptic mutations on the X chromosome in these crosses because the single-mutant *H3.3B^0^ / H3.3B^0^* homozygous females and *H3.3B^0^ / Y* hemizygous males were viable (data not shown). To determine if the adult female sterility is caused by impaired DNA replication or amplification, we labeled ovaries from the surviving double-mutant *H3.3* null females with the nucleotide analog EdU. H3.3 mutant ovaries had frequent follicle cell death and degeneration of midstage egg chambers, a stress response known as the vitellogenic checkpoint (Figure S4, B and C) (McCall 2004; Pritchett *et al.* 2009). This stress response resulted in fewer EdU-labeled H3.3 mutant than wild type follicle cells. However, the quality of EdU incorporation into follicle cell nuclei during mitotic cycle and endocycle S phases appeared similar between wild type and H3.3 mutant ovaries (Figure 6, A and B, and Figure S5, A and B).

To directly assess whether H3.3 is required for origin activity, we used EdU labeling to evaluate developmental gene amplification. Among the stage 10B-13 egg chambers that were produced in the H3.3 null females, incorporation of EdU into amplicon foci was similar between H3.3 null and wild type (∼15 egg chambers total over five biological replicates) (Figure 6, C and D, Figure S5, C and D, and Figure S6). The H3.3 null females also laid eggs with normal shells, further indicating that amplification of eggshell gene copy number is not greatly impaired (≥ five females tested for each of four biological replicates, data not shown). These results suggest that H3.3 is not essential for origin function during genomic DNA replication or developmental gene amplification.

## Discussion

We have investigated the function of H3.3 at origins using the unique methods afforded by the gene amplification system in *Drosophila*. Our results indicate that H3.3 is abundant at amplicon origins, consistent with the results of previous genome-wide studies that found a correlation between origins and H3.3 (Deal *et al.* 2010; MacAlpine *et al.* 2010; Eaton *et al.* 2011; Stroud *et al.* 2012). H3.3 was abundant at the DAFC-66D amplicon when replication initiation was blocked, and also early in oogenesis before the onset of amplification, indicating that the high level of H3.3 at this amplicon is not simply a result of deposition behind the replication forks during amplification. Importantly, however, H3.3 was not essential for origin function during amplification or genomic replication. These results resolve a long-standing question raised by genome-wide studies about the possible function of H3.3 at origins. Although not required, the high levels of H3.3 at origins suggest that it may reflect open chromatin attributes that are important for origin function.

Our data indicate that pre-RC binding sites at amplicon origins are enriched for H3.3—a property that is shared with origins that initiate genomic replication. Although H3.3 was not essential for amplicon origin function, it remains formally possible that it is required at a subset of other origins during genomic replication. It is known that cells license more origins than are used, and that dormant origins can initiate DNA replication during times of replication stress (Ge *et al.* 2007; Kawabata *et al.* 2011). Indeed, when we began this study, we considered that replication stress may contribute to the slow development and high degree of lethality of H3.3 null animals. The H3.3 null females had somewhat fewer EdU labeled follicle cells during genomic replication stages. This is likely an indirect effect of the high levels of cell death and egg chamber degeneration caused by the vitellogenic checkpoint response in midoogenesis because the quality of nuclear EdU labeling during genomic replication of H3.3 null cells appeared similar to wild type. The incorporation of EdU into amplicon foci also appeared similar between mutant and wild type, and the H3.3 null females produced eggs with normal shells. This observation is significant because it has been shown that amplification is a sensitized genetic system for detection of defects in proteins that are required for the function of all origins (Calvi 2006). For example, females homozygous for hypomorphic, missense alleles of the MCM6 subunit of the replicative helicase have normal genomic replication, but reduce amplification from 64-fold to 16-fold, which is manifest as undetectable amplicon foci in most cells, a severe thin eggshell, and female sterility (Schwed *et al.* 2002). It remains possible that H3.3 null females have a small decrease in final DNA copy number at the amplified loci, something we were unable to measure by qPCR because of the paucity of late stage egg chambers. The normal size and intensity of amplicon foci, however, suggest that there is not a major quantitative effect on origin efficiency. We favor the interpretation, therefore, that the oogenesis stress response in H3.3 null females is likely the result of the known effects of H3.3 deficits on transcription or chromatin maintenance, and that H3.3 is not required for the function of most origins (Sakai *et al.* 2009; Ray-Gallet *et al.* 2011; Szenker *et al.* 2011; Schneiderman *et al.* 2012).

In contrast to our results, other studies have shown that H3.3 is required for DNA replication at specific developmental times and during stress. In chicken DT40 cells, H3.3 is required for normal fork progression after UV irradiation, suggesting it may have a function at the fork during DNA repair or lesion bypass (Frey *et al.* 2014). Moreover, deficits in maternal H3.3, or its chaperone HIRA, result in DNA replication defects during the first zygotic S phase of mouse embryogenesis (Lin *et al.* 2014). Although we did not detect a difference in genomic replication or developmental amplification in H3.3 null animals, it appears that H3.3 may have a distinct requirement for DNA replication after DNA damage and during early development (Sakai *et al.* 2009; Lin *et al.* 2014; Wen *et al.* 2014).

One caveat to our results for H3.3 at amplicons is that H3.3A-GFP was overexpressed. This H3.3A-GFP transgene, however, is a validated system that has been used to show H3.3 chromatin deposition at other loci (Ahmad and Henikoff 2002; Schwartz and Ahmad 2005; Sakai *et al.* 2009). Our data support, therefore, that the amplicon origins have dynamic H3.3 exchange that is significantly higher than nonorigin loci.

Why is there a genome-wide correlation between H3.3 and origins? We favor the interpretation that this correlation reflects an open chromatin state that is conducive to pre-RC assembly. It is known that H3.3 nucleosomes have a dynamic association with DNA, and that their locations correspond to “nucleosome-depleted regions” (NDRs) because they are extracted under standard conditions for micrococcal nuclease (MNase) mapping (Jin and Felsenfeld 2007; Henikoff 2009; Jin *et al.* 2009). In fact, our recent MNase-Seq mapping in follicle cells indicated that the ORC binding sites ACE3 and ori-β at DAFC-66D are NDRs (Liu *et al.* 2015). Thus, the correlation between H3.3 and origins may reflect the ability of ORC to compete with dynamic H3.3-containing nucleosomes for binding to DNA. Our recent analysis also suggested, however, that ORC binds NDRs at DAFC-66D in part because it favors DNA sequences and structures that are disfavored by canonical nucleosomes, a result that may be generalizable genome-wide (Segal *et al.* 2006; Comoglio *et al.* 2015; Liu *et al.* 2015). Therefore, the correlation between ORC and H3.3 occupancy may reflect the fact that both are enriched at genomic regions with DNA sequences and structures that are disfavored by canonical nucleosomes (Ray-Gallet *et al.* 2011; Schneiderman *et al.* 2012; Comoglio *et al.* 2015; Liu *et al.* 2015).

It is important to stress that, while most ORC binding sites are NDRs and enriched for H3.3, many H3.3-containing NDRs are not ORC binding sites (Deal *et al.* 2010; Liu *et al.* 2015). This is further evidence that H3.3 is not instructive for specifying ORC binding sites and origins. It is clear that other chromatin attributes do have a major impact on origin function. For example, histone acetylation at DAFC-66D promotes ORC binding in stage 10 follicle cells and correlates with early-replicating and efficient origins during genomic replication (Aggarwal and Calvi 2004; Eaton *et al.* 2011). Both histone acetylation and ORC binding at DAFC-66D occurs over a ∼20 kb domain, with ORC binding likely occurring within the NDRs across the locus (Kim *et al.* 2011; Liu *et al.* 2012). Our ChIP-qPCR and immunofluorescent quantification indicated that H3.3 nucleosomes are also deposited over this same ∼20 kb domain, also likely within MNase defined “NDRs.” These data suggest, therefore, that this extended H3.3 distribution may be characteristic of a larger, open epigenome domain that is permissive for pre-RC assembly or activation during amplification. It will be interesting to determine whether this epigenome domain of nucleosome acetylation, ORC binding, and H3.3 reflects a change in higher order chromatin architecture that is conducive for origin function.

Our findings may also have broader relevance to understanding the pediatric gliomas that are caused by mutations in H3.3 and its chaperones (Schwartzentruber *et al.* 2012; Wu *et al.* 2012). If H3.3 also plays a minor role at mammalian origins, other functions of H3.3 should be emphasized to define the molecular mechanisms of those childhood cancers (Goldberg *et al.* 2010; Ray-Gallet *et al.* 2011; Schneiderman *et al.* 2012; Liu *et al.* 2014; Elsasser *et al.* 2015; Jang *et al.* 2015).

## Supplementary Material

Supplemental Material
