# Supplementary material for: The Histone Variant H3.3 Is Enriched at Drosophila Amplicon Origins but Does Not Mark Them for Activation
Source: G3 (Bethesda). 2016 Apr 6;6(6):1661–71. doi: 10.1534/g3.116.028068 (PMC4889662; doi:10.1534/g3.116.028068)
Supplement: Supplemental Material [file supp_6_6_1661__index.html]

Supplemental Material 

# The Histone Variant H3.3 Is Enriched at *Drosophila* Amplicon Origins but Does Not Mark Them for Activation

Supplemental Material for Paranjape and Calvi, 2016

Supplemental Material

**Files in this Data Supplement:**

- File S1 - Supplementary materials and methods. (.pdf, 71 KB)
- Table S1 - *p*-values for H3.3A-GFP occupancy in stage 10 (S10) follicle cells. (.pdf, 76 KB)
- Table S2 - *p*-values for the difference in H3.3A-GFP occupancy with or without expression of the CDK inhibitor Dacapo (Dap) in stage 10 (S10) follicle cells. (.pdf, 76 KB)
- Table S3 - *p*-values for H3.3A-GFP occupancy in stages 1-8 (S1-8) and stage 10 (S10) follicle cells. (.pdf, 79 KB)
- Table S4 - Location of ChIP-qPCR primers. (.pdf, 75 KB)
- Figure S1 - Quantification of H3-GFP and H3.3A-GFP intensity at the active DAFC-66D amplicon. (.pdf, 273 KB)
- Figure S2 - Amplification is inhibited by Dacapo. (.pdf, 128 KB)
- Figure S3 - ORC occupancy at DAFC-66D in stage 1-8 and stage 10 follicle cells. (.pdf, 142 KB)
- Figure S4 - A deficit of H3.3 induces a vitellogenic checkpoint response. (.pdf, 147 KB)
- Figure S5 - H3.3 is not essential for genomic DNA replication or developmental amplification. (.pdf, 152 KB)
- Figure S6 - EdU amplicon focus intensity in H3.3 null animals is similar to wild type. (.pdf, 192 KB)
