# Supplementary material for: The Histone Variant H3.3 Is Enriched at Drosophila Amplicon Origins but Does Not Mark Them for Activation
Source: G3 (Bethesda). 2016 Apr 6;6(6):1661–71. doi: 10.1534/g3.116.028068 (PMC4889662; doi:10.1534/g3.116.028068)
Supplement: Supplemental Material [file supp_g3.116.028068_FileS1.pdf]

File S1: Supplementary Materials and Methods

For Figure S1 C, ovaries from heat induced *HS-H3.3A-GFP* flies were incubated in 10µM EdU for 1 hour, then fixed with formaldehyde for 20 minutes. EdU was conjugated with Biotin-TEG-Azide (BT1085, Berry & Associates, Inc.) in a copper catalyzed Click-iT reaction, followed by detection using Cy-3 conjugated Avidin. α-GFP (rabbit, Molecular Probes, Life Technologies, USA) and secondary antibodies Alexa 488 anti- rabbit (Invitrogen) were used at 1:500 to label H3.3A-GFP. DNA was labeled with DAPI.
