# Supplementary material for: The Histone Variant H3.3 Is Enriched at Drosophila Amplicon Origins but Does Not Mark Them for Activation
Source: G3 (Bethesda). 2016 Apr 6;6(6):1661–71. doi: 10.1534/g3.116.028068 (PMC4889662; doi:10.1534/g3.116.028068)
Supplement: Supplemental Material [file supp_g3.116.028068_TableS1.pdf]

TABLE S1: *p*-values for H3.3A-GFP occupancy in stage 10 (S10) follicle cells

LOCUS[c]	*p*-value S10 at locus vs control[a]	*p*-value significance[b]
DAFC-66D – ACE -10	.0903	ns
DAFC-66D - a	.0078	**
DAFC-66D – ACE3	.0009	***
DAFC-66D - d	.0007	***
DAFC-66D - ori β	.0053	**
DAFC-66D - g	.0008	***
DAFC-66D – ACE +10	.0009	***
DAFC-7F	.0006	***
DAFC-22B	.0021	**
DAFC-30B	.0121	*
DAFC-34B	.0125	*
DAFC-62D	.0082	**
hsp70	.0016	**

a: The enrichment of H3.3A-GFP (as measured by % input) at the DAFCs was compared to its average enrichment at two negative control loci - 93E/F and 64A by Ratio Paired *t*-test.

b: not significant (ns), $p > 0.05$; ,* $p \leq 0.05$, ** $p \leq 0.01$; *** $p \leq 0.001$

c. Refer to Table S4 for primer locations.
