# Supplementary material for: The Histone Variant H3.3 Is Enriched at Drosophila Amplicon Origins but Does Not Mark Them for Activation
Source: G3 (Bethesda). 2016 Apr 6;6(6):1661–71. doi: 10.1534/g3.116.028068 (PMC4889662; doi:10.1534/g3.116.028068)
Supplement: Supplemental Material [file supp_g3.116.028068_TableS2.pdf]

TABLE S2: *p*-values for the difference in H3.3A-GFP occupancy with or without expression of the CDK inhibitor Dacapo (Dap) in stage 10 (S10) follicle cells

LOCUS[c]	p value S10 at locus in presence vs absence of Dap[a]	*p*-value significance[b]
DAFC-66D - ACE -10	.3213	ns
DAFC-66D - a	.6016	ns
DAFC-66D – ACE3	.7591	ns
DAFC-66D - d	.8109	ns
DAFC-66D - ori β	.8348	ns
DAFC-66D - g	.7364	ns
DAFC-66D - ACE +10	.5827	ns
DAFC-7F	.0514	ns
DAFC-22B	.7732	ns
DAFC-30B	.9587	ns
DAFC-34B	.7393	ns
DAFC-62D	.0410	*
hsp70	.2140	ns

a: Difference in enrichment of H3.3A-GFP (measured as % input) in stage 10 follicle cells was compared in the presence or absence of CDK inhibitor –Dap, by Ratio Paired *t*-test.

b: not significant (ns), $p > 0.05$; ,* $p \leq 0.05$

c. Refer to Table S4 for primer locations.
