# Supplementary material for: The Histone Variant H3.3 Is Enriched at Drosophila Amplicon Origins but Does Not Mark Them for Activation
Source: G3 (Bethesda). 2016 Apr 6;6(6):1661–71. doi: 10.1534/g3.116.028068 (PMC4889662; doi:10.1534/g3.116.028068)
Supplement: Supplemental Material [file supp_g3.116.028068_TableS3.pdf]

TABLE S3: *p*-values for H3.3A-GFP occupancy in stages 1-8 (S1-8) and stage 10 (S10) follicle cells

LOCUS[d]	*p*-value S1-8 at locus vs S1-8 control		*p*-value S1-8 at locus vs S10 at locus	
	p-value[a]	*p*-value significance[c]	*p*-value[b]	*p*-value significance[c]
DAFC-66D - ACE -10	.4509	ns	.5724	ns
DAFC-66D - a	.0118	*	.754	ns
DAFC-66D – ACE3	.0316	*	.0295	*
DAFC-66D - d	.0255	*	.0481	*
DAFC-66D - ori β	.0403	*	.2371	ns
DAFC-66D - g	.0281	*	0.0165	*
DAFC-66D - ACE +10	0.1574	ns	.036	*
DAFC-7F	.1345	ns	.0056	**
DAFC-22B	.0221	*	.2045	ns
DAFC-30B	.404	ns	.09	ns
DAFC-34B	.4961	ns	.037	*
DAFC-62D	.6255	ns	.0017	**
hsp70	.123	ns	.1235	ns

a: The enrichment of H3.3A-GFP (as measured by % input) in stage 1-8 at the DAFCs was compared to its average enrichment at two negative control loci - 93E/F and 64A by Ratio Paired *t*-test.

b. Difference in enrichment of H3.3A-GFP (measured as % input) in stage 1-8 vs stage 10 follicle cells by Ratio Paired *t*-test.

c: not significant (ns), $p > 0.05$; ,* $p \leq 0.05$, ** $p \leq 0.01$; *** $p \leq 0.001$

d. Refer to Table S4 for primer locations.
