# Supplementary material for: The Histone Variant H3.3 Is Enriched at Drosophila Amplicon Origins but Does Not Mark Them for Activation
Source: G3 (Bethesda). 2016 Apr 6;6(6):1661–71. doi: 10.1534/g3.116.028068 (PMC4889662; doi:10.1534/g3.116.028068)
Supplement: Supplemental Material [file supp_g3.116.028068_TableS4.pdf]

TABLE S4: Location of ChIP-qPCR primers.

Primer name	Primer Genomic Location[a]
ACE -10	3L: 8,715,901..8,716,007
DAFC-66D - a	3L: 8,725,294..8,725,393
ACE3	3L: 8,726,272..8,726,394
DAFC-66D - d	3L: 8,727,155..8,727,274
ori-β	3L: 8,728,136..8,728,249
DAFC-66D - d	3L: 8,729,741..8,729,825
ACE +10	3L: 8,736,262..8,736,368
DAFC- 22B	2L: 1,912,760..1,912,875
DAFC- 30B	2L: 9,544,399..9,544,491
DAFC- 34B	2L: 13,415,375..13,415,442
DAFC- 62D	3L: 2,272,717.. 2,272,790
DAFC- 7F	X: 8,482,316.. 8,482,431
64A	3L: 3,985,754..3,985,831
93E/F	3R: 21,695,975..21,696,058
hsp70	3R: 12,467,727..12,467,806

a: Coordinates for the primer pairs are based on *Drosophila melanogaster* reference genome Dmel_Release_6.09 (Flybase version FB2016_01)
