# Supplementary material for: The Histone Variant H3.3 Is Enriched at Drosophila Amplicon Origins but Does Not Mark Them for Activation
Source: G3 (Bethesda). 2016 Apr 6;6(6):1661–71. doi: 10.1534/g3.116.028068 (PMC4889662; doi:10.1534/g3.116.028068)
Supplement: Supplemental Material [file supp_g3.116.028068_FigureS1.pdf]

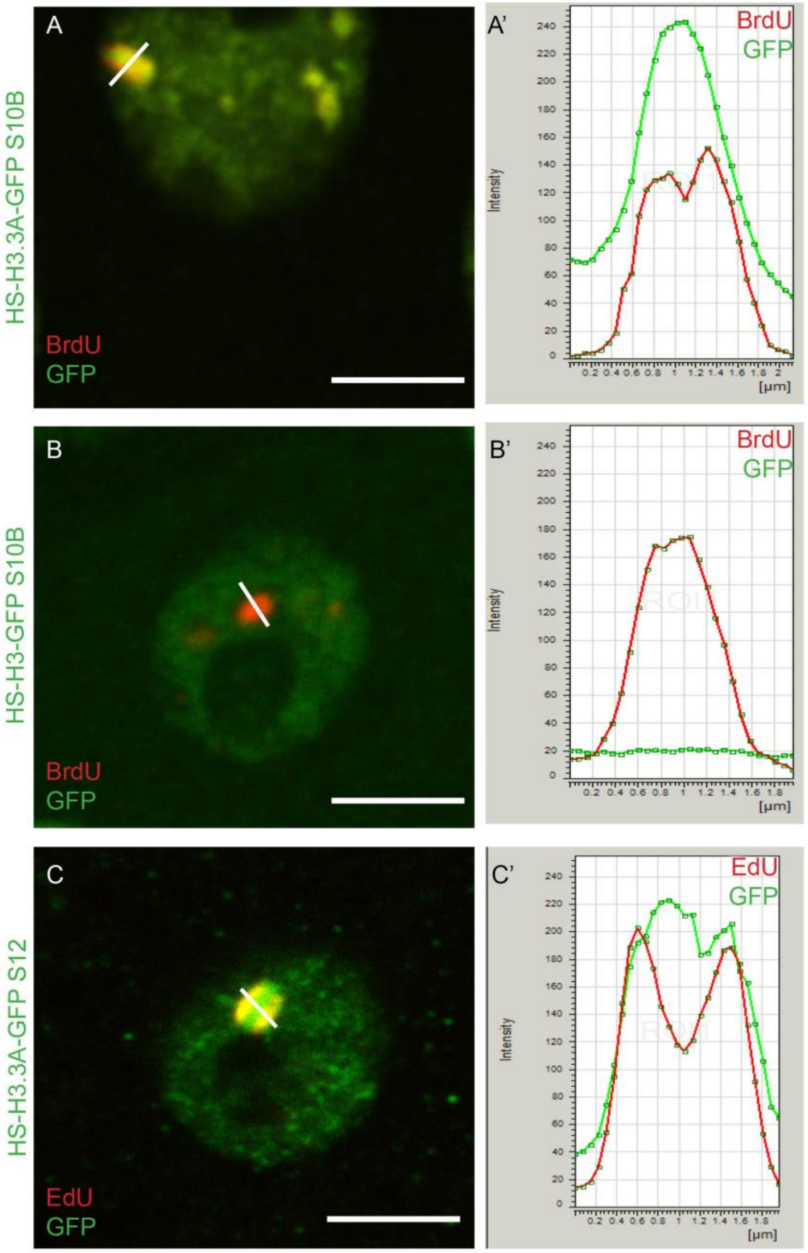

Figure S1: Quantification of BrdU and GFP intensity of *HS-H3-GFP* and *HS-H3.3A-GFP* at DAFC-66D.

(A,B) High magnification image of a stage 10B (S10B) follicle cell nucleus expressing H3.3A-GFP (green) (A) or H3-GFP (green) (B) with the highly-amplified DAFC-66D locus labeled by BrdU (red). C) A stage 12 follicle cell nucleus (S12) expressing H3.3A-GFP and DAFC-66D labeled with EdU, a stage when the ORC has departed, initiation has ceased, but forks continue to migrate bidirectionally outward (double bars). (A'-C') The intensity of GFP and BrdU/EdU double bars was quantified using Leica Advanced Fluorescence Lite software 2.6.0 build 7266. The region of interest (ROI) quantified is indicated by a white line in (A, B, C). Scale bars are 5µm.
