# Supplementary material for: The Histone Variant H3.3 Is Enriched at Drosophila Amplicon Origins but Does Not Mark Them for Activation
Source: G3 (Bethesda). 2016 Apr 6;6(6):1661–71. doi: 10.1534/g3.116.028068 (PMC4889662; doi:10.1534/g3.116.028068)
Supplement: Supplemental Material [file supp_g3.116.028068_FigureS2.pdf]

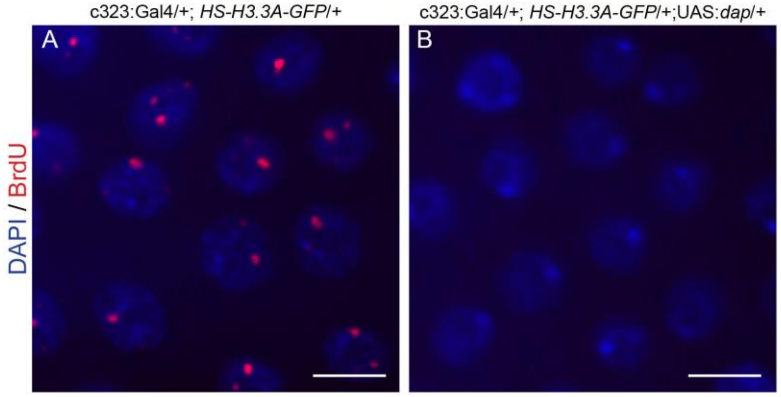

Figure S2: Amplification is inhibited by Dacapo.

DNA labeled with DAPI (blue) and BrdU incorporation (red) at the amplicon origins in stage 10 follicle cells expressing *HS-H3.3A-GFP* alone (A) or with the CDK inhibitor *dacapo* (*dap*) (B). Scale bars are 10 μm.
