# Supplementary material for: The Histone Variant H3.3 Is Enriched at Drosophila Amplicon Origins but Does Not Mark Them for Activation
Source: G3 (Bethesda). 2016 Apr 6;6(6):1661–71. doi: 10.1534/g3.116.028068 (PMC4889662; doi:10.1534/g3.116.028068)
Supplement: Supplemental Material [file supp_g3.116.028068_FigureS3.pdf]

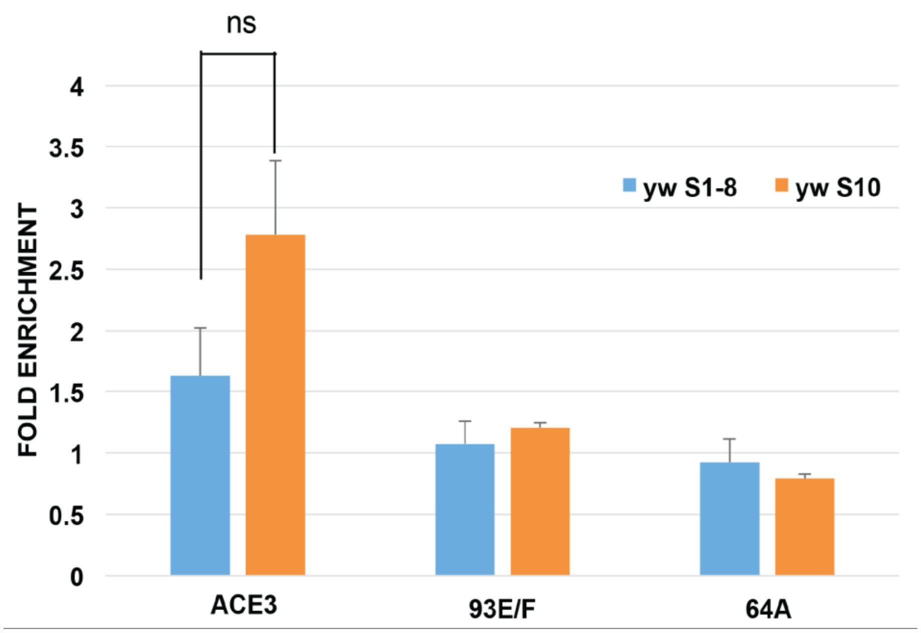


**Figure S3: ORC occupancy at DAFC-66D in stage 1-8 and stage 10 follicle cells**

Anti-Orc2 ChIP-qPCR analysis of stage 1-8 (S1-8) (blue bars) and stage 10 (S10) follicle cell nuclei (orange bars) from *y w* females. The *y*-axis represents fold enrichment of ORC at ACE3 of DAFC-66D relative to the two negative control loci 93E/F and 64A. The enrichment of ORC in early stage follicle cells was not significant (ns) when compared to ORC occupancy in stage 10 follicle cell nuclei or to the negative control loci in S1-8. Values represent average of two biological replicates and error bars represent range of values.
