# Supplementary material for: The Histone Variant H3.3 Is Enriched at Drosophila Amplicon Origins but Does Not Mark Them for Activation
Source: G3 (Bethesda). 2016 Apr 6;6(6):1661–71. doi: 10.1534/g3.116.028068 (PMC4889662; doi:10.1534/g3.116.028068)
Supplement: Supplemental Material [file supp_g3.116.028068_FigureS4.pdf]

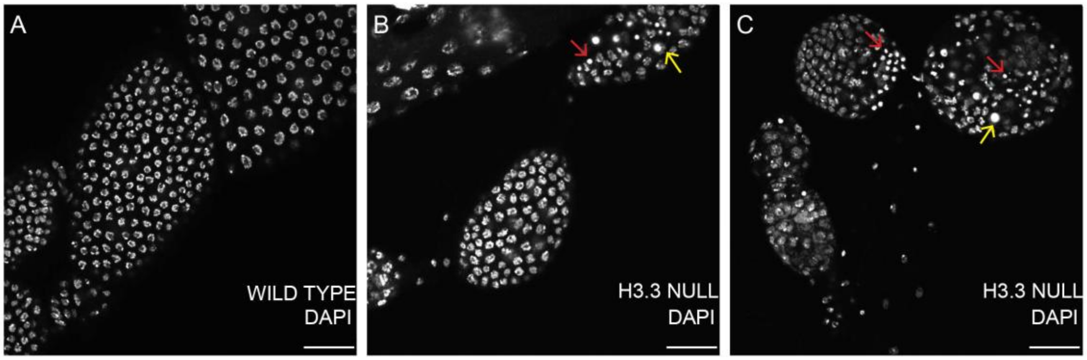

Figure S4: A deficit of H3.3 induces a vitellogenic checkpoint response.

Wild type (A) and H3.3 null ovaries (B, C) labeled with DAPI. In contrast to the wild type ovariole (A), both *H3.3B*^{o}; *H3.3A*^{2*1} / *Df(2L)BSC110* (B) *and H3.3B*^{o}; *H3.3A*^{2*1} / *Df(2L)Exel7022* null females (C) had frequent follicle cell death (indicated by red arrows in B and C), nurse cell death (indicated by yellow arrows in B and C), and degeneration of mid stage egg chambers.

Scale bars are 25 µm.
