# Supplementary material for: The Histone Variant H3.3 Is Enriched at Drosophila Amplicon Origins but Does Not Mark Them for Activation
Source: G3 (Bethesda). 2016 Apr 6;6(6):1661–71. doi: 10.1534/g3.116.028068 (PMC4889662; doi:10.1534/g3.116.028068)
Supplement: Supplemental Material [file supp_g3.116.028068_FigureS5.pdf]

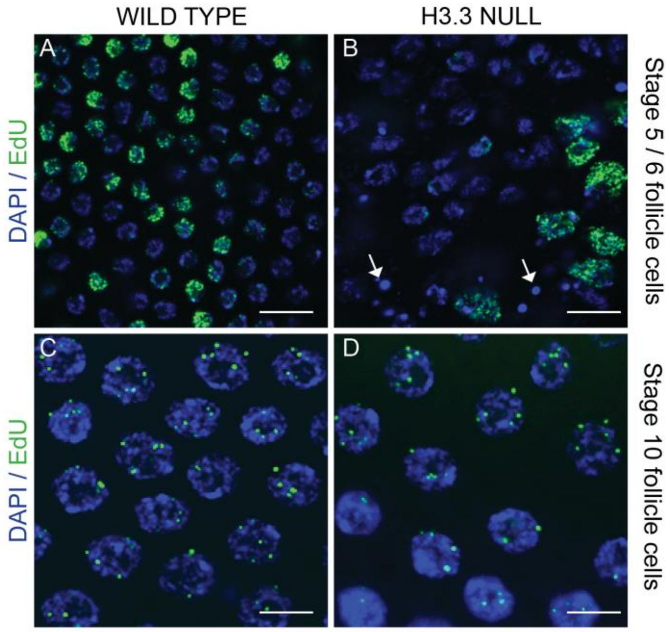

Figure S5: H3.3 is not essential for genomic DNA replication or developmental amplification.

(A-D) Follicle cell nuclei labeled with DAPI (blue) and EdU incorporation (green) during genomic replication (A, B) and amplification (C, D) in wild type control (A, C) or H3.3 null flies (B, D). Arrows in B point to two pycnotic nuclei in the H3.3 null mutant. These H3.3 null flies were homozygous mutant for an *H3.3B* null allele on the X and a null *H3.3A* allele over deficiency on the second, *H3.3B*^{o}; *H3.3A*^{2*1} / *Df(2L)Exel7022*. Scale bars are 10 µm.
