# Supplementary material for: The Histone Variant H3.3 Is Enriched at Drosophila Amplicon Origins but Does Not Mark Them for Activation
Source: G3 (Bethesda). 2016 Apr 6;6(6):1661–71. doi: 10.1534/g3.116.028068 (PMC4889662; doi:10.1534/g3.116.028068)
Supplement: Supplemental Material [file supp_g3.116.028068_FigureS6.pdf]

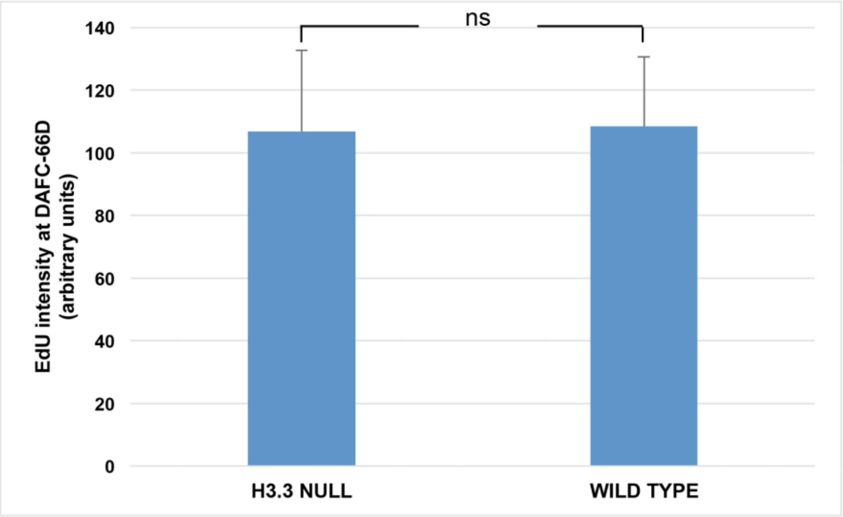

Figure S6: EdU amplicon focus intensity in H3.3 null animals is similar to wild type.

The fluorescence intensity of EdU incorporation at the DAFC-66D locus in stage 10B follicle cells was measured using image J. The *H3.3* null mutant female genotype was *H3.3B*^{o}; *H3.3A*^{2*1} / *Df(2L) BSC110.* The average fluorescence intensity and standard deviation is shown (n = 25 nuclei / sample). The difference between the two samples is not significant (n.s.) by a paired student's *t*-test.
